# The relationship between succession and intellectual capital with entrepreneurship in hospitals

**DOI:** 10.1186/s12913-023-09435-2

**Published:** 2023-05-11

**Authors:** Shoaib Rafiei, Mohammad Mehrtak, Mohammad Amerzadeh, Sima Rafiei, Saeideh Moosavi, Rohollah Kalhor

**Affiliations:** 1 Student Research Committee, School of Public Health, Qazvin University of Medical Sciences, Qazvin, Iran; 2 Healthcare Services Management, School of Medicine and Allied Medical Sciences, Ardabil University of Medical Sciences, Ardabil, Iran; 3 Social Determinants of Health Research Center, Research Institute for Prevention of Non-Communicable Diseases, Qazvin University of Medical Sciences, Qazvin, Iran

**Keywords:** Succession, Intellectual capital, Entrepreneurship, Qazvin, Iran

## Abstract

**Background:**

The hospital environment is very dynamic and faces many internal and external changes. Healthcare knowledge and technology are developing at a swift pace. This study investigated the relationship between succession and intellectual capital with entrepreneurship at the Qazvin University of Medical Sciences hospital, Iran.

**Methods:**

The number of employees working in six hospitals was 2256, and according to Morgan’s table, the required number of samples was 331. We distributed three hundred sixty-five questionnaires considering 10% of sample loss. We used a multi-stage stratified sampling method. In the first stage, each hospital was considered a stratum. After that, occupational groups were considered the next stratum within each hospital, and based on the ratio, the required number of samples for each occupational group was randomly selected. We used the Sobel test to investigate the mediating role of intellectual capital and the structural equation model to fit the research model.

**Results:**

Succession aspects, including culturalization, meritocracy, job promotion path, and the role of senior managers, have a positive and significant effect on intellectual capital. Succession is only effective on intellectual capital and does not affect the personnel’s entrepreneurship directly or through intellectual capital.

**Conclusion:**

Conducting training classes and intervention programs and using localized succession models can create a suitable platform for increasing organizational creativity and entrepreneurship, motivating the hospitals’ personnel, and increasing intellectual capital.

## Background

The environment of healthcare organizations, especially hospitals, is becoming more complex daily. People expect high-quality and safe services from hospitals. Policymakers and senior managers in the health system expect hospitals to provide high-quality services using fewer resources. The hospital environment is very dynamic and faces many internal and external changes. Healthcare knowledge and technology are developing at a swift pace. Therefore, managers and employees need to constantly improve their knowledge and skills to provide high-quality services [1, 2]. The right managers in the right units at the right time that do the right things to achieve results is necessary. Employing non-professional managers has a lot of adverse effects on the employees and the organization’s performance. The cost of keeping a managerial position vacant or choosing a weak manager is very high, and the hospital’s senior managers need to pay more attention to this issue. Hospitals face many challenges, such as the high cost of health services, high customer expectations, and drastic changes in political, economic, social, and technological issues. Managers are responsible for these challenges and need to provide high-quality health services at a low cost. With the development and implementation of the succession program, the time interval between the managers will be reduced [3, 4].

Succession is a tool to identify the current and future needs of the organization. Implementing succession programs in the hospital improves performance and achieves valuable results [5]. The succession in educational organizations is crucial because educational organizations’ output is input for other organizations. Organizational changes are unavoidable for various reasons, such as resignation, retirement, job promotion, or even death. If there is no systematic and planned solution, universities and educational institutions face issues such as leaving key posts empty or filling these posts with incompetent people [6].

Sustainable intellectual capital has three components: human capital, structural capital, and relational capital, which can help companies gain a competitive advantage [7]. The intellectual capital can lead to increasing profitability, improving the company’s strategic position, innovation, and unique technology, fulfilling standards, raising the credibility and image of the organization, increasing organizational reputation, reducing costs, increasing customer loyalty, improving service quality, and effectiveness and performance [8]. Human capital indicators are key employees’ professional and specialized competence, education, experience, the number of people with related backgrounds, and the exact distribution of customer responsibilities [9]. It can help the employees in optimal intellectual performance and improve the organization’s performance [10].

Intellectual capital is vast organizational knowledge that is unique to every organization and makes the organization constantly adapt to changing conditions. Therefore, since the hospital is vital in providing healthcare services, it can use intellectual capital for organizational value [11]. Applying intellectual capital can lead to innovation [12]. Organizations can benefit from management interventions that improve the organization’s intellectual capital to create approaches to innovation [13]. In organizations with intellectual capital, achieving high levels of growth and development is more accessible because organizations can use their competitive advantage indicators, including improving organizational innovation [14].

Entrepreneurship in the organization contributes significantly to the success and excellence of organizations. Entrepreneurial efforts in an organization with intellectual capital have become more effective and can provide competitive advantages and improve the company’s performance [15]. Entrepreneurship is a dynamic process of changing insight with innovation and creativity [16]. In countries like Iran, achieving competitive advantages and solving problems, such as unemployment, underdevelopment and entrepreneurship is necessary. Entrepreneurship plays three roles in every society: (1) It is the engine of economic development, strengthens economic growth and development, and (2) it increases the productivity of societies. (3) it creates technology, new products, and services. In terms of leadership, management, innovation, efficiency, job creation, competition, productivity, and the formation of new companies, entrepreneurs have an essential contribution to economic growth [17]. Nowadays, the opportunities and the number of entrepreneurial businesses in health services are increasing significantly due to extensive changes and developments [18]. Other studies in Iran show that intellectual capital has an impact on organizational entrepreneurship and organizational intelligence [19, 20]. A similar study found that the succession system is conducted based on the traditional approach, and managers are selected primarily based on a series of traditional measures such as the chief’s opinion and experience [21]. Therefore, this study aims to investigate the relationship between succession and intellectual capital with entrepreneurship in the Qazvin University of Medical Sciences, Iran hospitals.

## Methods

The current research is descriptive-analytical and cross-sectional. Since solutions can be presented and applied based on the results, it is also practical.

The population study in the first stage was all the employees (clinical, administrative, financial, and paraclinical) of public hospitals at the Qazvin University of Medical Sciences in Qazvin, Iran. There were six hospitals (Rajaei, Kosar, Bu Ali, Quds, Velayat, and 22 Bahman). The total number of employees working in medical training centers was 2256 people. According to Morgan’s table, the required number of samples was 331, and considering the 10% sample loss, we distributed 365 questionnaires according to the number of personnel in each hospital. We used a multi-stage stratified sampling method. In the first stage, each hospital was considered a stratum. After that, occupational groups were considered the next stratum within each hospital, and based on the ratio, the required number of samples for each occupational group was randomly selected. Rajaei, Kosar, Bu Ali, Quds, Velayat, and 22 Bahman hospitals had 68, 57, 92, 52, 77, and 19 samples, respectively. Finally, 313 questionnaires (86% response rate) were completed and submitted to the research team for analysis. The inclusion criteria were at least one year of work experience and willingness to participate in the study. The exclusion criteria were work experience of less than one year and unwillingness to participate in the study.

The questionnaire comprised four main parts. The first part was the demographic characteristics, and the second to fourth parts were the primary research questions to measure the variables. The first part of the questionnaire included gender, age, education, the field of study, organizational position, years of service, years of management, weekly working hours, and managerial level. The second part was related to succession planning, which included 16 closed questions from six components of planning, culturalization, systemic approach, meritocracy, job promotion path, and the role of senior managers on a Likert scale [22].

The third part contained 42 closed questions with three components: human capital, structural capital, and relational capital of (customer) staff and officials, with a five-point Likert scale [14].

The fourth part contained 12 questions. The components were reducing bureaucracy, change in employees’ behavior, strategic insight, and creating an energetic and supportive work environment with a 5-option Likert scale [23].

We examined the questionnaires’ validity through the face validity method. The questionnaires were given to eight management and entrepreneurship experts, who approved the questionnaires. We used Cronbach’s alpha method to measure the reliability. Thus, at acceptable levels, Cronbach’s alpha for succession, intellectual capital, and entrepreneurship questionnaires were 0.85, 0.81, and 0.80, respectively. The content validity and reliability of the succession questionnaire were 0.63 and 0.835 in another study in Iran [23]. The construct validity and reliability of the intellectual capital questionnaire were 0.67 and 0.84 in another Iranian study [24]. The content validity and reliability of the entrepreneur questionnaire were 0.85 and 0.90 in a similar study [25].

### Data analysis

We entered data into SPSS software. The most important technique used in this research was the Structural Equation Model (SEM). SEM is one of the most potent behavioral and social science analysis methods. It is a statistical method initially developed to model causal relationships between observed and latent variables. Since such issues are multi-variable, they cannot be analyzed with a two-variable method (where only one independent variable and one dependent variable are considered each time). Multivariate analysis refers to a series of analysis methods whose key feature is the simultaneous analysis of K-independent and N-dependent variables. Analysis of covariance structures, causal modeling, or SEM, is one of the primary methods of analyzing complex data structures. It is also used to analyze longitudinal and recent developments and extend its application to multi-level data and noncontinuous dependent variables. It is flexible in specifying a complex structure of random effects and in creating linear and non-linear constraints on the parameters [26–28]. Therefore, we used SEM since the current research had several independent variables. Pearson’s correlation coefficient was used to analyze data and test hypotheses. We used two statistical software: SPSS and AMOS23.

## Results

Cronbach’s alpha coefficient for succession, intellectual capital, and entrepreneurship questionnaires were 0.55, 0.81, and 0.80, respectively, proving the questionnaires’ reliability.

Among the 313 respondents, 213 (68.1%) were women. 241 (77%) were married. Forty people (12.8%) had a diploma, 191 (61%) had a bachelor’s degree, 69 (22%) had a master’s degree, and 13 people (4.2%) had a Ph.D.

Two hundred eight people (66.5%)were from the healthcare department, 80 people (25.6%) from the financial administration department, nine people (2.9%) from the technical-engineering department, eight people (2.6%) from the educational department, and eight people (2.6%) ) from other departments.

The average age of the participants was 36 (SD = 9), and the average employee’s experience was 12 years (SD = 9). They had management experience of an average of two years (SD = 2), and the average working hours per week was 49 h (SD = 16).

Table 1 shows the details of the leading research variables. It shows the number of questions for each questionnaire dimension, average score, and standard deviation. Total succession, intellectual capital, and entrepreneurship scores are 42.03, 121.99, and 32.

**Table 1:** Descriptive information on the three variables of succession, intellectual capital and entrepreneurship

Variable	Dimensions	Number of questions	Average	standard deviation
Succession	Planning	1–3	7.34	2.96
	Culturalization	4–7	10.55	3.44
	System Approach	8–9	5.29	1.75
	Meritocracy	10–12	8.07	2.84
	Job promotion path	13–14	5.47	1.88
	The role of senior managers	15–16	5.31	1.99
	The whole succession	1–16	42.03	13.34
Intellectual Capital	Human capital	2-5-7-9-11-13-17-18-24-25-26-31-33-34-37	42.73	9.60
	Structural capital	3-4-8-12-14-21-22-27-28-32-38-39-40	38.05	7.43
	Relationship capital (customer)	1-6-10-15-16-19-20-23-29-30-35-36-41-42	41.21	8.95
	Total intellectual capital	1–42	121.99	24.22
Entrepreneurship	Reduce bureaucracy	1	3	1
	Change in employee’s behavior	2-3-4-5-6	13.25	4.94
	Strategic approach	7-8-9-10	10.37	3.81
	Creating an energetic and supportive work environment	11–12	5.38	1.88
	The whole entrepreneurship	1–12	32	9.51

**Fig. 1:**
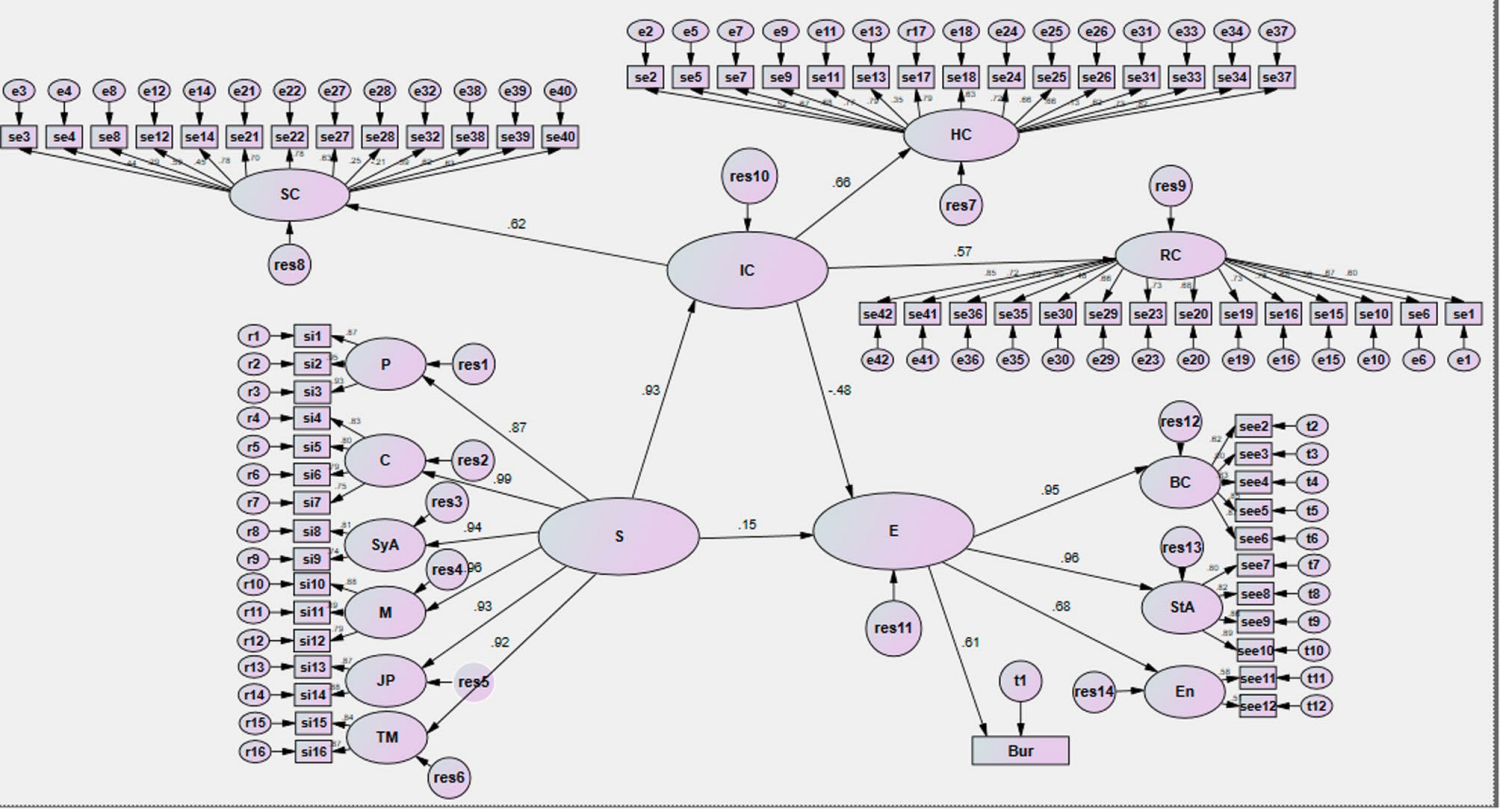
Estimation of the research model with non-standard coefficients. HC: Human Capital; RC: Relationship Capital; IC: Intellectual Capital; SC: Structural Capital; P: Planning; C: Culture; SyA: Systematic Approach; M: Meritocracy; JB: Job Promotion; TP: Top Managers; BC: Behavior; StA: Strategic Approach; E: Environment ; S: Succession; E: Entrepreneurship Bur: Bureaucracy

**Fig. 2:**
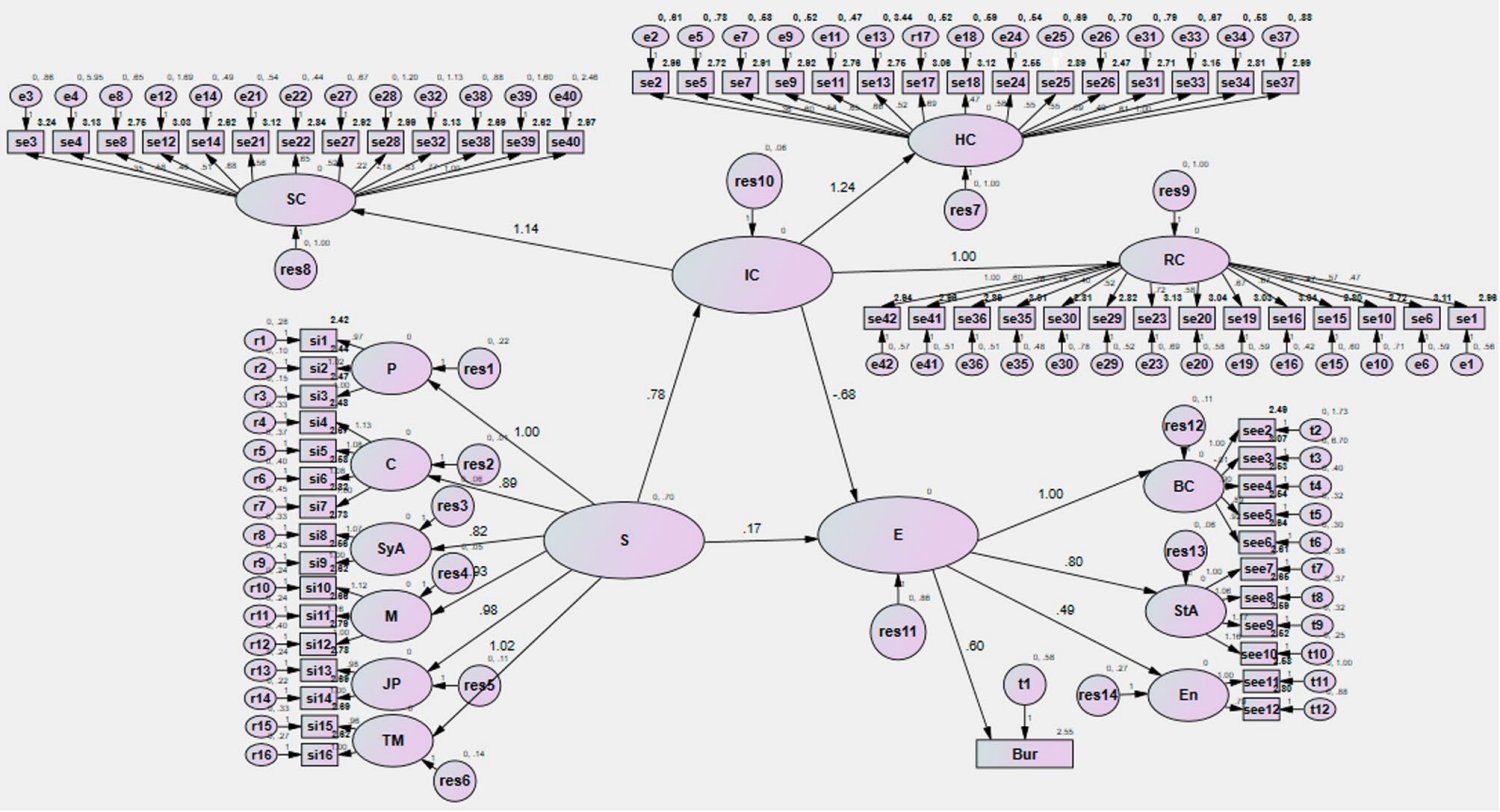
Estimation of the research model with standard coefficients. HC: Human Capital; RC: Relationship Capital; IC: Intellectual Capital; SC: Structural Capital; P: Planning; C: Culture; SyA: Systematic Approach; M: Meritocracy; JB: Job Promotion; TP: Top Managers; BC: Behavior; StA: Strategic Approach; E: Environment ; S: Succession; E: Entrepreneurship Bur: Bureaucracy

Figures 1 and 2 show the path coefficient between the model variables on each path corresponding to the research assumptions. This standard path coefficient is a number between 1 and − 1. The positive sign indicates a positive relationship between the research variables. For example, a path coefficient of 0.93 has been obtained between succession and intellectual capital. (p < 0.05) According to the study’s findings, no significant relationship was observed between succession and entrepreneurship and intellectual capital and entrepreneurship (P > 0.05).

We used the value of the t-Student statistic corresponding to each coefficient to measure the significance of the estimated paths, reported in Table 2.

**Table 2:** Regression weights in SEM parameters

Hypothesis symbol	Hypothesis			Standard path coefficient	Non-standard path coefficient	standard error	Statistics T (meaning number)	P	The result of the hypothesis
1	Succession	<---	Intellectual Capital	0.932	0.777	0.0840	9.247	***	proving the hypothesis
2	Succession	<---	Entrepreneurship	0.148	0.174	0.484	0.359	0.719	Failure to confirm the hypothesis
3	Intellectual Capital	<---	Entrepreneurship	-0.484	-0.684	0.618	-1.107	0.268	Failure to confirm the hypothesis
4	Meritocracy	<---	Succession	0.959	0.932	0.060	15.509	***	proving the hypothesis
5	Senior Managers	<---	Succession	0.915	1.022	0.061	16.666	***	proving the hypothesis
6	Job promotion	<---	Succession	0.928	0.982	0.057	17.209	***	proving the hypothesis
7	Culturalization	<---	Succession	0.994	0.887	0.059	14.952	***	proving the hypothesis
8	Planning	<---	Succession	0.872	1.000				
9	Human Capital	<---	Intellectual Capital	0.655	1.242	0.160	7.785	***	proving the hypothesis
10	Relationship capital	<---	Intellectual Capital	0.573	1.000				
11	Structural capital	<---	Intellectual Capital	0.624	1.142	0.159	7.195	***	proving the hypothesis

The value of the t statistic for the path coefficients was over 1.96, except for the second hypothesis (succession - entrepreneurship) and the third (intellectual capital - entrepreneurship), which were not significant (P > 0.05). Therefore, all path coefficients were significant except paths two and three.

To fit the factor model obtained from the previous step, chi-square indices, Goodness of Fit Index (GFI (, Root Mean Square Error of Approximation (RMSEA), Comparative Fit Index (CFI), and Normal Fit Index were used. Table 3 shows the fit indices obtained from the model.

**Table 3:** Fit indices of the conceptual model

index name	Limit	The amount obtained
$( \frac{{\varvec{\chi }}^{2}}{\varvec{d}\varvec{f}}$	Less than 3	2.242
**GFI**	Above 0.9	0.918
**RMSEA**	Less than 0.08	0.068
**CFI**	Above 0.9	0.904
**NFI**	Above 0.9	0.939

According to the above table, the values obtained for the indicators: Chi-do, GFI, RMSEA, CFI, and NFI were within the defined range. Therefore, the fit of the obtained model at this stage indicates a perfect fit.

## Discussion

This study aimed to evaluate the effect of succession on entrepreneurship with the mediating role of intellectual capital among teaching hospital personnel at the Qazvin University of Medical Sciences, Iran.

The mean of succession in this study was 42.03, which is almost in the average range and is consistent with the average of succession in the studies of Najibi et al., Davoudi and Yaqoubi, and Mehrtak et al. [23, 29]. It shows the weakness of educational hospitals in using succession. Considering the level of succession in this study, using a comprehensive localized development program for employees and planning to reach the desired level of competence reduces the problems in implementing succession. The institutionalization of a comprehensive succession program also leads to improving the efficiency and effectiveness of the processes and eventually improving the health level of society.

The average intellectual capital in this study was 121.99, at the average range, while in two studies, it was reported above the average level [30, 31]. Considering the dynamics of the environment, organizations need to identify, measure and value their intellectual capital to adapt to the conditions and compete with their competitors. The organization will create a competitive advantage by paying attention to capital and intangible assets such as human, structural, and relationship assets.

The average entrepreneurship score in this study was 32, which is in the average range, while in another study, it was above the average level [32]. It seems that there is no specific program in hospitals for developing entrepreneurship. Entrepreneurship is one of the development tools because the entrepreneurial people in the organization create a platform for the organization’s success. Every organization needs a proper structure and entrepreneurial people to cultivate spontaneous and innovative people. Managers have a significant role and can cause the emergence of entrepreneurial behaviors and characteristics in employees.

Our results showed that succession significantly affects intellectual capital, and it was at an average level, which is in line with Hassasi and Ahmadi, Manteghi et al., Burqani Farahani et al., Broumand and Tavousi, Shahin Arang and Ayas, and Dostdar et al. findings [33–38]. Succession is a process to ensure the continuity of leadership in key positions and develop intellectual capital and knowledge for the future. It originates from the human resource planning strategy. It makes organizations turn their vision inside the organization and adopt succession strategies that focus on developing and cultivating their existing talents [39, 40]. Therefore, supervisors and managers need to plan for succession by forming a specialized task group and providing the ground for the growth and excellence of the organization’s human and structural capital.

This study’s second hypothesis is that succession significantly affects entrepreneurship, which was rejected. Samei and Faiz Bakhsh’s findings were not consistent with our results. The difference might be due to differences in organizational culture and context. It is crucial for the successor to have the necessary competencies to continue the development of entrepreneurship in organizations, so one of the managers’ duties is to use appropriate methods to empower the successor. In previous research, no specific study was on fostering an entrepreneurial approach [41]. According to the senior managers of the companies under study, passing training courses and learning modern science and technology has led to a better understanding of issues and familiarity with innovative solutions, technologies, and products. In addition, attending management training courses has also helped them to acquire knowledge and general management skills. These abilities have helped the successor make better entrepreneurial decisions, especially in the organization’s new business [42].

The third hypothesis of this study was that intellectual capital has a significant effect on entrepreneurship, which was rejected. This study’s results are inconsistent with the Taqvai Yazdi et al. Also, the results of the third hypothesis of the present study were inconsistent with Hosseini et al. (2013), Ramzanpour Nargesi et al.), and Ebrahimian and Khalilpour (2013). Also, in foreign studies, the results of Pearson et al. (2015), Fan (2015), Fan and Zhou (2012), and Chahal Bakshi (2014) were different from our findings ([35, 43–49]. Other studies in Iran also showed a significant relationship between intellectual capital and entrepreneurship [50, 51]. Our results differ from other studies because other organizations’ context is different from ours. According to the studies, the human component, as the most critical pillar of intellectual capital in the organization, includes qualifications, technical and knowledge skills of employees and their level of experience, creativity, innovativeness of people, communication skills, and teamwork of employees. Each can influence organizational entrepreneurship differently, but these results differed from our findings.

## Conclusion

The study results showed that succession has a significant relationship with intellectual capital. However, intellectual capital has no significant relationship with entrepreneurship or succession with entrepreneurship. This study’s average variables score shows no specific program in hospitals for developing intellectual intelligence, entrepreneurship, and succession. According to the research findings about the creation of entrepreneurship, and the infrastructure for using creative and entrepreneurial forces in teaching hospitals, we recommend conducting training classes and intervention programs. Identifying the dimensions of intellectual capital in health service providers can create a suitable platform for increasing organizational creativity and entrepreneurship. Localized succession models can motivate the hospitals’ personnel and increase intellectual capital and creativity.

### Rigor of study

We only studied public hospitals, and private hospitals were not considered in this study. Non-cooperation of some employees in completing the questionnaire was another point.

## Data Availability

The datasets used and/or analyzed during the current study available from the corresponding author on reasonable request. The entire dataset is in Farsi language. The Data can be available in English language for the readers and make available from the corresponding author on reasonable request.
